# Transcatheter aortic valve implantation in the first 500 patients: a single-center retrospective study

**DOI:** 10.3325/cmj.2024.65.424

**Published:** 2024-10

**Authors:** Marko Noc, Ales Pleskovic, Maja Rojko, Hrvoje Reschner, Natasa Cernic, Branko Cveticanin, Matjaz Span, Stamenko Susak, Rok Stopar, Danijel Petrovic, Naomi Ana Noc, Ana Bosnjak, Nenad Danojevic, Miladin Djordjevic, Metka Zorc

**Affiliations:** 1 MC Medicor International Center for Cardiovascular Diseases, Izola, Slovenia; 2 Faculty of Medicine, University of Ljubljana, Ljubljana, Slovenia; 3 General Hospital Izola, Izola, Slovenia

## Abstract

**Aim:**

To determine the procedural characteristics, results, and long-term outcomes of the first 500 consecutive patients undergoing transcatheter aortic valve implantation (TAVI) at the MC Medicor International Center for Cardiovascular Diseases Izola (Slovenia).

**Methods:**

Data were collected from the institutional registry. The date of death was obtained from the National BIRPIS system by using the patient’s health insurance card number. The difference in 30-day mortality was assessed between two consecutive cohorts of 250 patients, patients who received self-expandable (SEV) and those with balloon-expandable (BEV) valves, and between patients ≤80 and >80 years old.

**Results:**

Between December 2016 and September 2023, 500 patients (80 ± 6 years, 52% men, EuroScore II, 4.09 ± 4.11), including 3.2% with degenerated surgical prosthesis, underwent TAVI. After predilatation (57%), SEV was implanted in 87.5% and BEV in 12.5% of the patients. The mean postprocedural gradient was 10 ± 4 mm Hg, with more than moderate regurgitation in 0.4%. Emergency cardiac/vascular surgery was performed in 1.4%, and stroke occurred in 0.8%. The new permanent pacemaker (PPM) rate decreased from 19% to 7% (*P* < 0.001) in the second cohort, and the mean postprocedural transaortic gradient was significantly lower after SEV compared with BEV (9 ± 4 vs 13 ± 4 mm Hg; *P* < 0.001). There was no difference in 30-day mortality between the first and second cohort of 250 patients (1.2% vs 1.2%; *P* = 1.000), cohorts of 50 patients from number 0 to 500 (0% vs 2.0%; *P* = 0.391), SEV and BEV groups (0.9% vs 1.6%; *P* = 0.487), and patients ≤80 and >80 years old (2.0% vs 0.4%; *P* = 0.119).

**Conclusion:**

TAVI results in our study are comparable with international standards. PPM rate decreased over time, and postprocedural gradient was lower after SEV. Learning curve, type of valve, and patient age did not affect 30-day mortality.

Transcatheter aortic valve implantation (TAVI) has become the preferred treatment of symptomatic aortic stenosis regardless of patient age and risk of open-heart surgery (1-3). TAVI, as a percutaneous procedure allowing for rapid rehabilitation, yields comparable or even better results than surgical aortic valve replacement. At the MC Medicor International Center for Cardiovascular Diseases (Izola, Slovenia), the TAVI program started in December 2016, and the results of the first 109 patients were published in 2020 (4). In the present study, we assessed the procedural characteristics, results, and long-term outcomes of the first 500 consecutive patients. We addressed the likelihood of 5-year survival, as well as the subgroup of patients receiving “valve in valve” (ViV) due to degenerated surgical prosthesis. Furthermore, we also investigated the impact of our TAVI learning curve, valve type, and patient age on 30-day mortality.

## Patients and methods

Our study enrolled consecutive patients undergoing TAVI at the MC Medicor International Center for Cardiovascular Diseases (Izola, Slovenia) between December 12, 2016 and September 17, 2023. Data were obtained from the prospective institutional registry of percutaneous cardiovascular interventions. The study was approved by the National Medical Ethics Committee. All patients received a comprehensive explanation of the TAVI procedure, after which they signed an informed consent. The consent form also included an agreement to enter clinical data into the institutional registry.

The selection of patients by the Medicor heart team, preprocedural analyses, procedural features, post/procedural treatment, and follow-up were previously reported (4). In brief, self-expandable (SEV, Evolut R/Pro, Medtronic, Minneapolis, MN, USA) or balloon expandable (BEV, SAPIEN 3, Edwards Lifescience, Irvine, CA, USA) valves were used. The valve type and size were selected based on the protocolized TAVI computed tomography analysis (CTA) and the patient’s characteristics. Coronary angiography and percutaneous coronary intervention were performed as staged procedures before TAVI. For implantation of SEV, coplanar projection of three cusps was used until December 11, 2019, when right-left cusp-overlap projection, together with commissural alignment, became a default strategy (5). For BEV implantation, standard coplanar projection of three cusps was used. In patients undergoing ViV, single or double coronary protection was used in case of the high likelihood of coronary occlusion, which was estimated by CTA (6). Intentional leaflet laceration to prevent TAVI-induced coronary obstruction (BASILICA) was not used (7). Echocardiography was performed on an ambulatory basis before TAVI, immediately after valve implantation in the catheterization laboratory and before hospital discharge. After hospital discharge, patients were followed by the Medicor outpatient cardiology unit.

### Statistical analysis

The normality of distribution was assessed with a Kolmogorov-Smirnov test. Numerical data are presented as mean values with standard deviations, and categorical data as absolute numbers and percentages. Numerical data were compared with an unpaired *t* test or Mann-Whitney U test. For comparison of categorical variables, a χ^2^ test or Fisher exact test was used. The date of eventual death after TAVI was obtained from the National BIRPIS system using the patient’s health insurance card number. The likelihood of five-year survival was estimated with the Kaplan-Meier curve, with October 1, 2023 as the last observational date. A *P* value of less than 0.05 was considered significant. The analysis was performed with SPSS, version 22 (IBM Corp, Armonk, NY, USA).

## Results

The average age was 80 years, and the mean EuroScore II was 4.09 (Table 1). The mean left ventricular ejection fraction (LVEF) was 58%, and 32% of patients had concomitant obstructive coronary disease. The mean transaortic gradient was 47 mm Hg, and the calculated aortic valve area was 0.9 cm^2^.

**Table 1:** Patient characteristics and features of aortic stenosis in 500 consecutive patients undergoing transcatheter aortic valve implantation (TAVI) at the MC Medicor International Center for Cardiovascular Diseases, Izola (Slovenia)*^†^

Patient characteristics	
Age, years	80 ± 6 (60-95)
Men, n (%)	258 (52)
Logistic EuroScore	16.12 ± 12.82 (1.66-80.44)
EuroScore II	4.09 ± 4.11 (0.69-50.13)
STS score	2.83 ± 2.17 (0.38-16.02)
Left ventricular ejection fraction, %	58 ± 11 (20-80)
Concommitant obstructive coronary disease, n (%)	162 (32)
Post PCI/CABG, n (%)	141 (28)
Permanent pacemaker, n (%)	34 (7)
Aortic stenosis features	
Maximal gradient, mmHg	75 ± 17 (33-128)
Mean gradient, mmHg	47 ± 11 (18-84)
Aortic valve area, cm^2^	0.9 ± 0.3 (0.3-1.2)
Bicuspid valve, n (%)	22 (4.4)
Degenerated surgical valve, n (%)	16 (3.2)

*Abbreviations: STS – Society of Thoracic Surgeons Score for hospital mortality; PCI – percutaneous coronary intervention; CABG – coronary artery bypass grafting.

†Data are presented as mean values ± standard deviations or medians and ranges, unless otherwise indicated.

**Figure 1:**
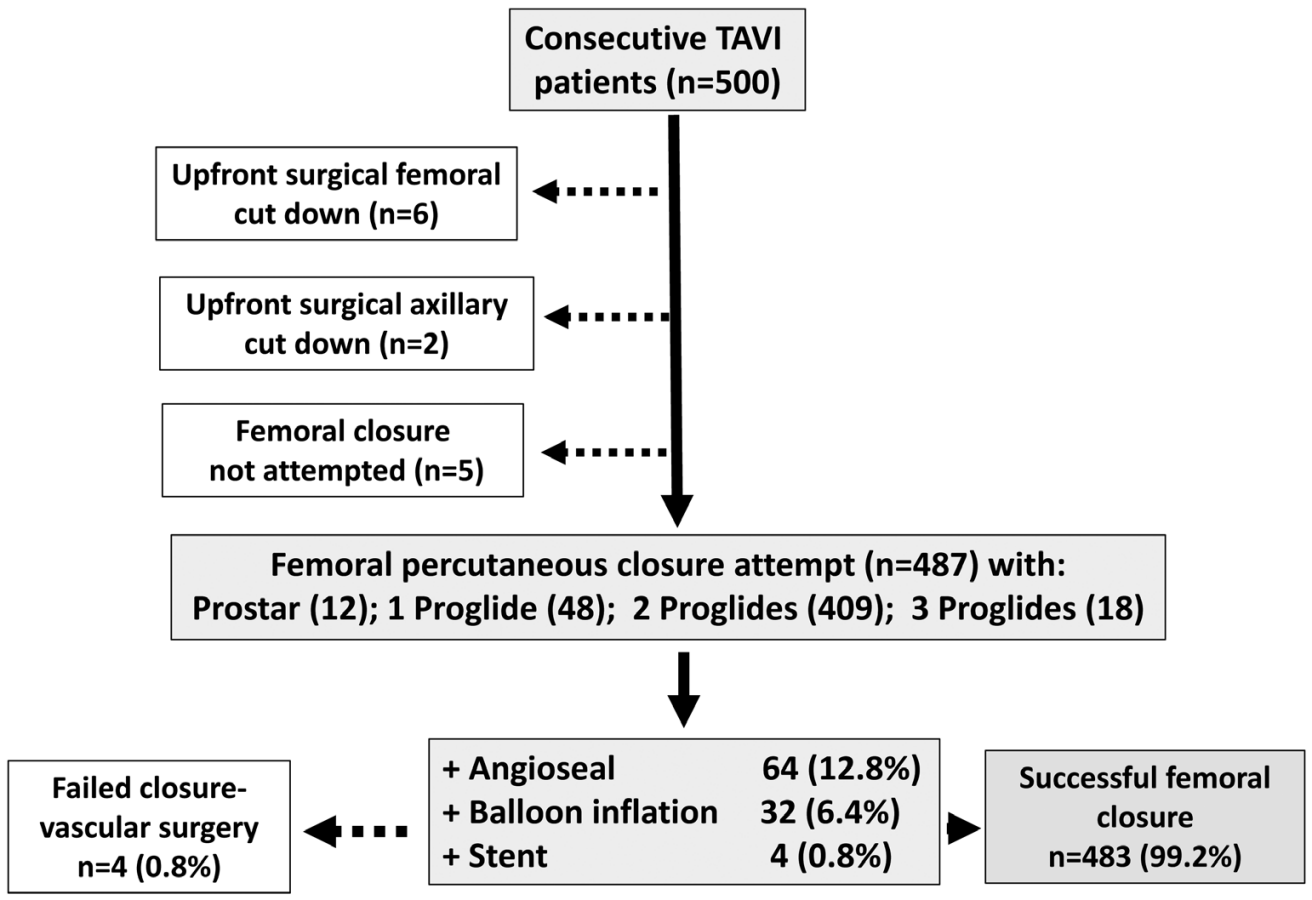
Femoral closure after transcatheter aortic valve implantation (TAVI) using either Prostar XL (Abbott, Lake Forest, IL, USA) or ProGlide (Abbott, Lake Forest, IL, USA) suture-mediated closure system followed by additional Angioseal VIP vascular closure device (Terumo, Somerset, NJ, USA), balloon inflation, or peripheral stent.

Conscious sedation was used in 91% of the patients, with percutaneous femoral access in 99% (Table 2). After balloon predilatation (57%), SEV was implanted in 87.5% of the patients, and BEV in 12.5%. The mean postprocedural transvalvular gradient was 10 mm Hg, and more than moderate aortic regurgitation was documented in 0.4% of the patients. Percutaneous closure, which was successful in 99.2%, was primarily attempted by either Prostar XL or ProGlide (both from Abbott, Lake Forest, IL, USA) suture-mediated closure systems. In case of incomplete hemostasis, additional Angioseal VIP plug closure device (Terumo, Somerset, NJ, USA), balloon inflation, or peripheral stent implantation via contralateral femoral access were used (Figure 1).

**Table 2:** Procedural characteristics and outcomes in 500 consecutive patients undergoing transcatheter aortic valve implantation (TAVI) at the MC Medicor International Center for Cardiovascular Diseases, Izola (Slovenia)*

Procedural characteristics	n (%)
Conscious sedation, n (%)	455 (91)
Arterial access, n (%)	
percutaneous femoral	495 (99)
upfront femoral surgical cut down	3 (0.6)
left axillary surgical cut down	2 (0.4)
Balloon predilatation, n (%)	284 (57)
Successful valve deployment, n (%)	498 (99.6)
SEV (Medtronic Evolut R/Pro)	436 (87.5)
BEV (Edwards Sapien 3)	62 (12.5)
Valve postdilatation, n (%)	93 (19)
Coronary protection, n (%)	13 (3)
**Procedural results**	
Mean gradient, median±SD (range); mmHG	10 ± 4 (3-26)
>Moderate aortic regurgitation	2 (0.4)
New permanent pacemaker implantation	65 (13)
30-day mortality	6 (1.2)

*Abbreviations: SEV – self-expandable valve; BEV – balloon-expandable valve.

**Figure 2:**
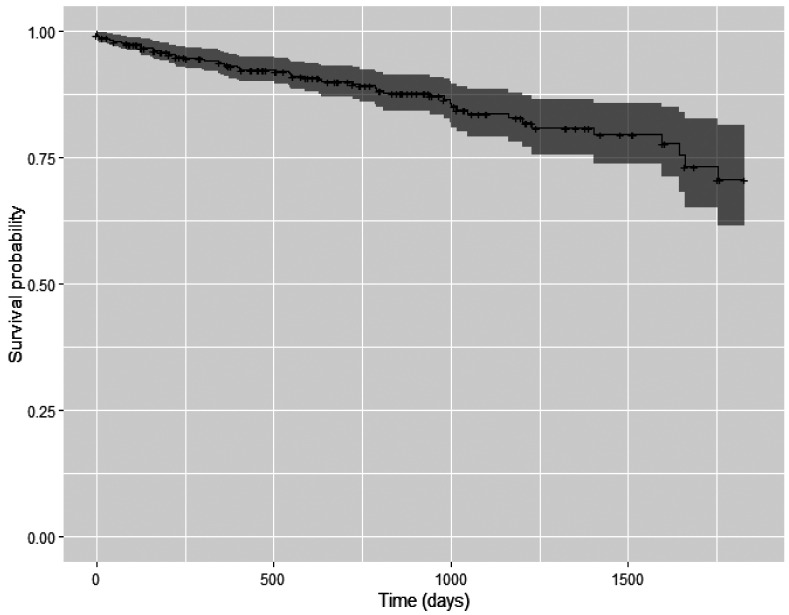
The Kaplan-Meier estimate of the likelihood of five-year survival in 500 consecutive patients undergoing transcatheter aortic valve implantation (TAVI) at the MC Medicor International Center for Cardiovascular Diseases, Izola (Slovenia).

Major periprocedural complications included stroke (0.8%), right or left ventricle perforation (1.0%), aortic injury (0.8%), rupture of the common iliac artery (0.2%), and acute left main occlusion (0.2%). Emergency pericardiocentesis was performed in 0.8%, unprotected left main stenting in 0.2%, and cardiac/vascular surgery in 1.4% of the patients (Table 3). A new permanent pacemaker (PPM) was implanted in 13% of the patients. The 30-day mortality was 1.2% (95% CI 0.4%-2.5%), one-year mortality 6.6% (95% CI 4.1%-8.9%), two-year mortality 10.3% (95% CI 7.1%-13.4%), three-year mortality 16.2% (95% CI 11.4%-20.7%), four-year mortality 20.4% (95% CI 14.1%-26.2%), and five-year mortality 29.2% (95% CI 18.6%-38.4%) (Figure 2).

**Table 3:** Major periprocedural complications and emergency interventions in 500 consecutive patients undergoing transcatheter aortic valve implantation (TAVI) at the MC Medicor International Center for Cardiovascular Diseases, Izola (Slovenia)

Complications	n (%)
Cerebrovascular insult	4 (0.8)
Left/right ventricle perforation	5 (1.0)
Annular/aortic rupture	2 (0.4)
Aortic dissection type A	1 (0.2)
Infrarenal aortic dissection	1 (0.2)
Rupture of the common iliac artery	1 (0.2)
Acute left main occlusion	1 (0.2)
**Emergency interventions**	
Pericardiocentesis	4 (0.8)
Left main stenting	1 (0.2)
Cardiac surgery	3 (0.6)
Vascular surgery	4 (0.8)
Hospital stay, mean ±SD (range); days	6 ± 3 (1-36)

Sixteen of 500 patients (3.2%) underwent ViV due to degenerated Mitroflow (n = 7), Trifecta (n = 3), Perceval (n = 2), Mosaic (n = 1), Biocor (n = 1), or Solo Freedom (n = 1), or after CARVAR valve-sparing surgery (n = 1). The predominant mechanism of valve failure was severe aortic stenosis with the mean transvalvular gradient of 46 ± 15 mm Hg, except in 4 patients who presented with severe aortic regurgitation. Following right (n = 2), left (n = 2), or double (n = 6) coronary protection with guiding catheter, guidewire, and undeployed stent, Evolut R/Pro was implanted in 15 patients (94%) and Sapien 3 in 1 patient (6%). Valve fracturing/remodeling using a non-compliant balloon was performed in 10 patients (62.5%) followed by the deployment of protective coronary stents in each instance. Mean transaortic gradient decreased from 46 ± 15 mm Hg to 16 ± 5 mm Hg (*P* < 0.001) without significant aortic regurgitation. None of the patients died during the index hospitalization or follow-up.

We then divided our patients into the first and second cohort of 250 patients (Table 4). The second cohort had a lower logistic EuroScore, the American Society of Thoracic Surgeons (STS) score, and the incidence of obstructive coronary disease and previous revascularization. The second cohort more frequently underwent conscious sedation (86% vs 96%), valve predilatation (32% vs 81%), and >1 recapture with SEV (44% vs 24%). The second cohort had a lower PPM rate (7% vs 19%) and a shorter hospital stay (5 vs 7 days) than the first cohort. There was no difference in 30-day mortality between the cohorts (1.2% vs 1.2%; *P* = 1.00). Also, no significant difference in 30-day mortality, which ranged between 0% and 2%, was observed between the cohorts of 50 patients from number 0 to 500 (*P* = 0.391).

**Table 4:** Statistically significant differences between the first and second cohort of 250 patients at the MC Medicor International Center for Cardiovascular Diseases, Izola (Slovenia)*

Parameter	First cohort (n = 250)	Second cohort (n = 250)	P
Logistic EuroScore, mean±SD	17.63 ± 13.94	14.60 ± 11.42	0.004
STS score, mean±SD	2.92 ± 2.06	2.75 ± 2.27	0.029
Obstructive coronary disease, n (%)	92 (37)	70 (28)	0.036
Post PCI/CABG, n (%)	84 (34)	57 (23)	0.007
Conscious sedation, n (%)	216 (86)	239 (96)	<0.001
Valve predilatation, n (%)	81 (32)	203 (81)	<0.001
>1 Recapture (Evolut R/Pro), n (%)	61 (24)	111 (44)	<0.001
New permanent pacemaker, n (%)	47 (19)	18 (7)	<0.001
Hospital stay, mean±SD (days)	7 ± 4	5 ± 2	<0.001

*Abbreviations: STS – Society of Thoracic Surgeons Score for hospital mortality; PCI – percutaneous coronary intervention; CABG – coronary artery bypass grafting.

Patients undergoing SEV implantation had significantly increased preprocedural transaortic gradients and decreased postprocedural gradients than patients undergoing BEV (9 ± 4 vs 13 ± 4 mm Hg; *P* < 0.001) (Table 5). There were no significant differences in 30-day mortality between the groups (0.9% vs 1.6%; *P* = 0.487).

**Table 5:** Statistically significant differences in the parameters listed in Tables 1-3 between self-expandable (Medtronic, Evolut R/Pro) and balloon-expandable valve (Edwards, Sapien 3) at the MC Medicor International Center for Cardiovascular Diseases, Izola (Slovenia)

Parameter	Evolut R/PRO (n = 436)	Sapien (n = 62)	P
Maximal gradient, mean±SD; mmHg	75 ± 17	69 ± 16	0.001
Mean gradient, mean±SD; mmHg	47 ± 11	43 ± 11	0.001
Valve postdilatation, n (%)	92 (21)	1 (2)	<0.001
Mean postprocedural gradient, mean±SD; mmHg	9 ± 4	13 ± 4	<0.001

Patients >80 years old had significantly increased logistic EuroScore, EuroScore II, and STS score compared with patients ≤80 years, but the groups did not significantly differ in 30-day mortality (0.4% vs 2.0%; *P* = 0.119) (Table 6).

**Table 6:** Statistically significant differences in the parameters listed in Tables 1-3 between patients aged ≤80 years vs >80 years at the MC Medicor International Center for Cardiovascular Diseases, Izola (Slovenia)*

	≤80 years (n = 247)	>80 years (n = 253)	P
Age, mean±SD (years)	75 ± 4	85 ± 3	<0.001
Men, n (%)	140 (57)	118 (47)	0.025
Logistic EuroScore, mean±SD	12.86 ± 11.40	19.30 ± 13.34	<0.001
Euroscore II, mean±SD	3.35 ± 3.06	4.81 ± 4.83	<0.001
STS Score, mean±SD	2.06 ± 1.23	3.59 ± 2.58	<0.001
Hospital stay, mean±SD (days)	5 ± 2	6 ± 4	0.004
30-day mortality, n (%)	5 (2.0)	1 (0.4)	0.119

*Abbreviations: STS – Society of Thoracic Surgeons Score for hospital mortality.

## Discussion

In the present study, we extended our preliminary observation in 109 patients undergoing TAVI (4) to more comprehensively address the procedural characteristics, results, and long-term outcomes in a large cohort of consecutive 500 patients with wide distribution in age (60-95 years) and procedural risk (EuroScore II 0.69-50.13). Our results regarding postprocedural transaortic gradient, the incidence of significant aortic regurgitation, and 30-day mortality are comparable with the pivotal randomized trials and internationally accepted standards of care (1-3,8). Moreover, the five-year mortality of our patients is essentially the same as in the recently published NOTION randomized trial, which enrolled very similar patients with a mean age of 79 years and STS score 3.0 (9). This is also true for our complication rate, including the rate of stroke (0.8%) and the need for emergency cardiac/vascular surgery (1.4%), which were fortunately exceptional. Due to the predominant use of the sheathless implantation of the lowest profile valve (Evolut R/Pro), requiring only a 5.0-mm diameter of iliofemoral arteries, we were able to use percutaneous femoral access in 99% of patients. Moreover, careful preprocedural CTA analysis of the common femoral artery enabled arterial puncture at the site with minimal calcium load, thereby facilitating percutaneous closure in more than 99% of the patients.

We separately addressed ViV patients, in more than 60% of whom we achieved acceptable postprocedural transaortic gradient by using valve fracturing/remodeling with a non-compliant balloon. Even though our ViV experience was limited to only 16 patients, 63% of them had degenerated surgical valves with an increased likelihood of acute coronary occlusion after TAVI. Instead of the complex technique known as BASILICA (7), we used upfront coronary protection with stenting after valve deployment (6). Importantly, despite left main and/or right coronary ostial stenting with significant protrusion into the sinus Valsalva in >50% of the patients, there were no deaths during the follow-up. This finding indirectly indicates preserved long-term stent patency despite interaction with the valve frame.

The analysis of cohorts of 250 patients showed no significant TAVI learning curve that would harm the outcome of our patients. Although the first cohort of 250 patients had a higher risk profile, it did not differ in 30-day mortality compared with our second cohort. This was also true for the consecutive cohorts of 50 patients from number 0 to 500. The most likely explanation for the acceptable results already at the beginning of the program is our proctoring period provided by two internationally recognized experts, which was extended up to the patient number 90. This undoubtedly improved our skills and the ability to predict, prevent, and treat complications. On the other hand, the PPM rate significantly decreased in the second cohort of 250 patients (from 19% to 7%), which is acceptable considering that 85% of patients received SEV. We believe that a decreased PPM rate is predominantly related to an improved technique and confidence regarding SEV implantation, together with a very early adoption of right-left cusp overlap projection allowing higher valve implantation (5).

We further compared the results between SEV and BEV cohorts. Patients with SEV, despite having a higher preprocedural transaortic gradient, had a 4 mm lower postprocedural gradient. This is a well-known finding, which is primarily related to the supra-annular SEV design leading to a larger aortic orifice area (8). Although a 4 mm greater transaortic gradient after BEV did not increase 30-day mortality, it is unknown if such difference may lead to more valve degeneration at a longer follow-up.

We also compared our TAVI patients according to age, taking 80 years as a separator. As expected, patients ≥80 years had significantly increased preprocedural risk scores, which indicates more comorbidities. Despite this, 30-day mortality was low and comparable between the cohorts. This was surprising since some centers have recently reported up to 5.5% mortality at 30 days despite comparable age and EuroScore II (10).

When interpreting the results of our study, it should be kept in mind that our study was single center, retrospective, and mostly descriptive. Accordingly, the results cannot be generalized to other institutions because of specific features related to the selection of patients, TAVI procedure, evaluation of the results, and patient follow-up.

In conclusion, TAVI results at the MC Medicor International Center for Cardiovascular Diseases in Izola are comparable with the currently accepted international standards in terms of patient selection, procedural features, and long-term outcomes. Medicor has thereby undoubtedly contributed to an improved medical care of patients with symptomatic aortic stenosis in Slovenia.

## References

[1] Leon MB Smith CR Mack MJ Makkar RR Svensson LG Kodali SK Transcatheter or surgical aortic-valve replacement in intermediate-risk patients. N Engl J Med 2016 374 1609 20 10.1056/NEJMoa1514616 27040324

[2] Mack MJ Leon MB Thourani VH Makkar R Kodali SK Russo M Transcatheter aortic-valve replacement with a balloon-expandable valve in low-risk patients. N Engl J Med 2019 380 1695 705 10.1056/NEJMoa1814052 30883058

[3] Reardon MJ Van Mieghem NM Popma JJ Kleiman NS Sondergaard L Mumtaz M Surgical or transcatheter aortic-valve replacement in intermediate-risk patients. N Engl J Med 2017 376 1321 31 10.1056/NEJMoa1700456 28304219

[4] Pleskovic A Rojko M Cernič Suligoj N Cvetičanin B Span M Petrovic D Results of transcatheter aortic valve implantation (TAVI) in the international centre for cardiovascular diseases MC Medicor. Zdrav Vestn 2021 90 139 49

[5] Mendiz OA Noc M Fava CM Gutiérrez Jaikel LA Sztejfman M Pleskovič A Impact of cusp-overlap view for TAVR with self-expandable valves on 30-day conduction disturbances. J Interv Cardiol. J Interv Cardiol 2021 2021 9991528 10.1155/2021/9991528 34007249 PMC8099519

[6] Noc M Cveticanin B Kar S Mendiz AO Left main protection and emergency stenting during TAVR with self-expandable valve. J Struct Heart Dis 2018 4 240 24 10.12945/j.jshd.2018.008.18

[7] Kitamura M Majunke N Holzhey D Desch S Bani Hani A Krieghoff C Systematic use of intentional leaflet laceration to prevent TAVI-induced coronary obstruction: feasibility and early clinical outcomes of the BASILICA technique. EuroIntervention 2020 16 682 90 10.4244/EIJ-D-20-00386 32597392

[8] Thiele H Kurz T Feistritzer HJ Stachel G Hartung P Eitel I Comparison of newer generation self-expandable vs. balloon-expandable valves in transcatheter aortic valve implantation: the randomized SOLVE-TAVI trial. Eur Heart J 2020 41 1890 9 10.1093/eurheartj/ehaa036 32049283

[9] Thyregod HGH Jørgensen TH Ihlemann N Steinbrüchel DA Nissen H Kjeldsen BJ Transcatheter or surgical aortic valve implantation: 10-year outcomes of the NOTION trial Eur Heart J 2024Feb 7:ehae043 10.1093/eurheartj/ehae043 38321820 PMC10984572

[10] Kolar T Lakič N Kotnik A Štubljar D Fras Z Bunc M Similar clinical outcomes with transcatheter aortic valve implantation and surgical aortic valve replacement in octogenarians with aortic stenosis. Front Cardiovasc Med 2022 9 947197 10.3389/fcvm.2022.947197 36386346 PMC9640378

